# Supplementary material for: Enhanced Photoluminescence and Photocatalytic Efficiency of La-Doped Bismuth Molybdate: Its Preparation and Characterization
Source: Materials (Basel). 2019 Dec 20;13(1):35. doi: 10.3390/ma13010035 (PMC6981540; doi:10.3390/ma13010035)
Supplement: Supplementary file 1 [file materials-13-00035-s001.pdf]

materials

Supplementary **Material**

Enhanced Photoluminescence and Photocatalytic Efficiency of La-Doped Bismuth Molybdate: Its Preparation and Characterization

Muhammad Waqar [1], Muhammad Imran [1,*], Syed Farooq Adil [2,*], Sadia Noreen [1], Shoomaila Latif [3], Mujeeb Khan [2] and Mohammed Rafiq H. Siddiqui [2]

1 Institute of Chemistry, Punjab University Lahore-Pakistan, Lahore 54590, Pakistan waqar0100@gmail.com (M.W.); sadian543@gmail.com (S.N.)
2 Department of Chemistry, King Saud University, Riyadh 11451, Kingdom of Saudi Arabia; kmujeeb@ksu.edu.sa (M.K.); rafiqs@ksu.edu.sa (M.R.H.S.)
3 Department of Chemistry, University of Lahore-Pakistan, Lahore 54590, Pakistan; shoomaila_latif@yahoo.com
* Correspondence: imran_inorganic@yahoo.com (M.I.); sfadil@ksu.edu.sa (S.F.A.)

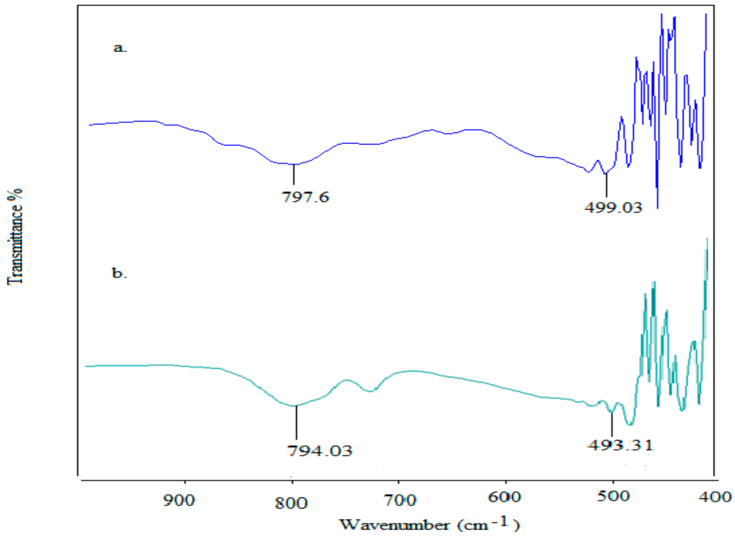

Figure S1. FT-IR spectra of Bi_2MoO_6 and La-doped Bi_2MoO_6 nanocomposite.
